# Dim Light Exposure and Myopia in Children

**DOI:** 10.1167/iovs.18-24415

**Published:** 2018-10

**Authors:** Erica G. Landis, Victoria Yang, Dillon M. Brown, Machelle T. Pardue, Scott A Read

**Affiliations:** 1 Neuroscience, Emory University, Atlanta, Georgia, United States; 2 Department of Ophthalmology, Emory University, Atlanta, Georgia, United States; 3 Atlanta Veterans Affairs Medical Center, Atlanta, Georgia, United States; 4 Biomedical Engineering, Georgia Institute of Technology, Atlanta, Georgia, United States; 5 School of Optometry and Vision Science, Queensland University of Technology, Brisbane, Queensland, Australia

**Keywords:** myopia, scotopic, photopic, light exposure, children, refractive error

## Abstract

**Purpose:**

Experimental myopia in animal models suggests that bright light can influence refractive error and prevent myopia. Additionally, animal research indicates activation of rod pathways and circadian rhythms may influence eye growth. In children, objective measures of personal light exposure, recorded by wearable light sensors, have been used to examine the effects of bright light exposure on myopia. The effect of time spent in a broad range of light intensities on childhood refractive development is not known. This study aims to evaluate dim light exposure in myopia.

**Methods:**

We reanalyzed previously published data to investigate differences in dim light exposure across myopic and nonmyopic children from the Role of Outdoor Activity in Myopia (ROAM) study in Queensland, Australia. The amount of time children spent in scotopic (<1–1 lux), mesopic (1–30 lux), indoor photopic (>30–1000 lux), and outdoor photopic (>1000 lux) light over both weekdays and weekends was measured with wearable light sensors.

**Results:**

We found significant differences in average daily light exposure between myopic and nonmyopic children. On weekends, myopic children received significantly less scotopic light (*P* = 0.024) and less outdoor photopic light than nonmyopic children (*P* < 0.001). In myopic children, more myopic refractive errors were correlated with increased time in mesopic light (*R* = −0.46, *P* = 0.002).

**Conclusions:**

These findings suggest that in addition to bright light exposure, rod pathways stimulated by dim light exposure could be important to human myopia development. Optimal strategies for preventing myopia with environmental light may include both dim and bright light exposure.

Myopia, or nearsightedness, is a significant public health concern in many developed and developing countries. It is estimated that by 2050, approximately 50% of the world population will be affected by myopia.^1^ This raises concerns about a growing population with increased risk of high myopia and associated visually debilitating disorders later in life, including glaucoma, retinal detachment, and cataracts. One strategy to combat this rise in myopia is to prevent its onset in childhood. Behaviors that protect against myopia or slow the progression of myopia are of particular interest, as they can be implemented at the population level and would not involve pharmaceutical interventions in children.

Behavioral comparisons of children with and without myopia have identified time spent outdoors as a risk factor for both the presence of myopia and the progression of axial elongation (see Ref. 2 for review). The first study to identify this correlation analyzed myopia progression of school children in Finland and found increased time outdoors correlated with decreased progression.^3,4^ Subsequently, multiple studies confirmed that myopic children spent less time outdoors than nonmyopic children, or that time outdoors negatively correlated with myopia development.^5–12^ Multifactorial analyses have suggested that bright light exposure during time outdoors, over other factors like physical activity, was the most likely candidate for driving the protection against myopia.^2,10,13–15^

These findings changed our understanding of how environmental conditions during development could alter the prevalence and severity of myopia. However, most of these studies relied on questionnaire data, usually answered by parents who could be influenced by recall bias. Objective studies of the association between light exposure, not just time outdoors, and myopia are therefore needed to more comprehensively understand these environmental impacts, and to evaluate what amount of bright light is necessary for myopia prevention. In 2015, Read et al.^16^ used wearable light sensors (the Actiwatch, a wrist watch style device capable of measuring personal ambient light exposure every 30 seconds) to assess the daily light exposure patterns of myopic and nonmyopic children, and to examine the relationship between light exposure and longitudinal changes in axial eye growth. When children aged 10 to 15 had their light exposure assessed over two, 14-day periods in one year, it was observed that myopic children spent significantly less time in light >1000 lux than nonmyopic children, and a significant association between slower axial eye growth and greater time spent in light >3000 lux was found. By using objective personal light exposure measures, in addition to questionnaires, the authors could make more specific conclusions about the timing and brightness of light that might be necessary to slow or prevent myopia progression.

The focus on bright light exposure as a preventive measure against myopia has been well supported by human and animal studies.^17–22^ In animal models of experimental myopia in which single factors are more easily controlled, bright light exposure was also shown to be protective during myopia development (Siegwart JT, et al. *IOVS* 2012;53:ARVO E-Abstract 3457).^17,18,20,23^ Importantly, randomized and controlled clinical intervention studies in children have replicated this finding. Children who were assigned to spend daily recess in a gymnasium, getting physical activity without outdoor light, were more likely to develop myopia over a 1-year period than children who spent the same amount of time outside.^21^ Additionally, two clinical trials that administered additional outdoor time to children during the school day showed decreased onset and development of myopia compared with the control groups.^22,24^ However, most of these studies did not use objective methods to assess personal daily light exposure, so we cannot know the full range of light that children were exposed to during the day.

Studies have shown that the portion of the day children spend in bright light (greater than 1000 lux) is relatively small, roughly 1 to 2 hours on weekdays, even for nonmyopic children.^11,16,25–28^ Thus, many hours of each day fit into a general category of “less than bright” exposure, including indoor light and dim light. Evidence that dim light exposure specifically could be important for refractive eye growth has been found in several animal models. First, a previous study by our group has shown that illuminance levels of 0.005 lux, similar to starlight, can prevent lens-induced myopia in a mouse model (Landis E. *IOVS* 2015;56:ARVO E-Abstract 2152). Additionally, mice with dysfunctional rod photoreceptors have no response to form deprivation myopia compared with wild-type animals, indicating rod-driven vision may be essential for detecting the visual input needed for correct refractive eye growth.^29^ In animals with foveas, the peripheral retina is dominated by rod photoreceptors compared with the cone-rich fovea, and has been implicated in the development of myopia. When myopiagenic inputs are projected to the peripheral retina of rhesus monkeys or chickens, with the central retina either ablated by laser or given normal vision, myopic refractions and elongated axial lengths are observed.^30–34^ In humans, myopia progression is slowed in children treated with contact lenses that reduce peripheral hyperopia.^35^ However, the relative contributions of the peripheral and central retina are still debated, and it is unknown if these protective inputs are due to rod versus cone stimulation to the retina or optical consequences.^36–41^

The implications that dim light exposure may modulate myopia development through rod activity leads to the question of how much dim light exposure children typically receive and whether the patterns of dim light exposure differ between myopic and nonmyopic children. To address this gap in knowledge, we used the ROAM study light exposure data set first published by Read et al.^16^ to analyze the amount of dim light to which myopic and nonmyopic children are typically exposed.

## Methods

### Participants and Data Collection

For this experiment, data were collected as described previously.^16,25^ Briefly, Actiwatch-2 Activity Monitors (Philips, NV, USA) were worn by 102 children between 10 and 15 years of age from the Brisbane area in Queensland, Australia, who were enrolled in the Role of Outdoor Activity in Myopia (ROAM) study. The Queensland University of Technology human research ethics committee approved all study procedures before data collection began and all parents and children gave written, informed consent. All participants were treated according to the guidelines set by the Declaration of Helsinki. At the beginning of the study, the refractive error of all children was determined by noncycloplegic subjective refraction aiming for maximum plus/least minus for best visual acuity. No participant had a history of ocular disease. All the children exhibited best corrected visual acuity of logMAR 0.00 or better in each eye. Each child was classified as either myopic (average noncycloplegic spherical equivalent subjective refractive error [SER] from right and left eyes of −0.50 DS of myopia or more, with at least one eye exhibiting −0.75 DS or more myopia) or nonmyopic (average SER from right and left eyes between < +1.25 and > −0.50, with neither eye exhibiting −0.75 DS or more myopia). Forty children were classified as myopic with a mean SER of −2.39 ± 1.50 DS, and each myopic child was paired with a nonmyopic child (mean SER of 0.34 ± 0.30 DS) of the same sex and similar age, who wore the Actiwatch device over the exact same period as the matched myopic child. Classification as either myopic or nonmyopic did not change for any of the children throughout the first 12 months of the study. One pair of participants were excluded during follow-up visits due to the development of ocular pathology in one nonmyopic child. Therefore, data from 80 children were included in the final analysis presented here. A small group of older nonmyopic children (*n* = 20) also participated in the study but wore the Actiwatch device at a different time compared with the matched myopic and nonmyopic children. This group was excluded from our analyses to ensure that daily and seasonal variations in dim light exposure did not influence the findings.

Baseline ocular measurements were taken between May and November of 2012 with objective measures of light exposure taken by an Actiwatch over the following 14-day period. A second 14-day period of light exposure measurements was conducted 6 months later. Therefore, light exposure measurements were spread across seasons, and all were collected during the school term. Ocular measurements were taken at the initial visit, 6 months later, and again 1 year after the initial measurements were taken.

The Actiwatch is a wristwatch style device that contains a silicon photodiode light sensor capable of measuring visible light within 400 to 900 nm (peak sensitivity 570 nm). The light sensors were programmed to record illuminance data every 30 seconds, resulting in approximately 80,640 measurements per child. The sensitivity of the Actiwatch at dim illuminance levels was measured by comparison with a calibrated luxmeter (Extech HD450 Datalogging light meter; Extech Instruments, Waltham, MA, USA) across a range of 16 dim (<50 lux) light levels. The Actiwatch sensor showed high agreement with the luxmeter (mean difference 2.1 ± 1.1 lux), indicating a high level of sensitivity for assessing dim light levels (Supplementary Fig. S1). Each participant completed an activity diary that was used to estimate illuminance during times when the Actiwatch was removed (e.g., for sports practice or bathing). If the watch was removed for more than 90% of any day, that day was eliminated from analysis. This resulted in an average of 23.5 ± 0.34 days per participant over both collection periods. Data from both collection periods were included and combined in this analysis, as there were no significant differences in light exposure or time spent awake between the two periods. Only data taken during waking hours was used; this was determined by the Actiwatch sleep and wake detection algorithms and the activity diaries.^42–44^ Myopic and nonmyopic children spent equal amounts of time awake per day (myopic: 14.73 ± 1.75 hours, nonmyopic: 14.62 ± 1.63 hours; *t*-test = 0.046, df = 78, *P* = 0.9632; Table).

**Table:**
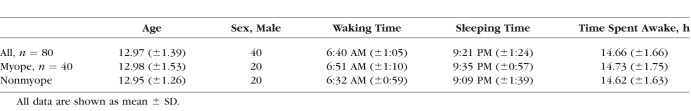
Demographics and Sleep Patterns of Myopic and Nonmyopic Children in the ROAM Study Cohort

### Data Analysis

All data cleaning and analysis were done using R and R Studio (https://cran.r-project.org) or Python for SPSS (IBM Corp., New York, NY, USA). Once recordings had been cleaned for missing data as described above and measurements taken during sleeping hours were eliminated, the light exposure data for each subject was binned into four different light intensity levels: scotopic light (<1–1 lux), mesopic (>1–30 lux), indoor photopic (>30–1000 lux), and outdoor photopic (>1000 lux). These light bins were chosen to provide an overview of the children's habitual light exposure patterns across a variety of conditions, with a particular focus on the dimmer end of the light exposure spectrum. This also allowed us to isolate the light that would be activating rod pathways in the retina. To analyze the typical light exposure patterns of myopic and nonmyopic children across the day, the time in each light level per half hour after waking each day was averaged for both groups of participants across each of the four light levels. Total time spent in each light level each day was averaged for myopic and nonmyopic children and compared. For these analyses, data were also divided into weekdays (Monday–Friday) and weekends (Saturday–Sunday) to reflect the differences in behavior across the week. Significant differences in these patterns were assessed using analyses of covariance (ANCOVAs) with Tukey post hoc comparisons, treating time as a third-order covariate due to its nonlinear relationship with light over the day. Significance for these comparisons was set at *P* < 0.03.^45^ All reported interactions with time (refraction × time or time x day) are on the quadratic term. Correlations between refractive error in myopic and nonmyopic children and the time spent in each light bin per day were determined through Pearson correlation analysis. The refractive error of myopic participants in this study were not normally distributed. Therefore, in myopic children these correlations were performed on refractive error transformed by taking the cube root.

## Results

**Figure 1:**
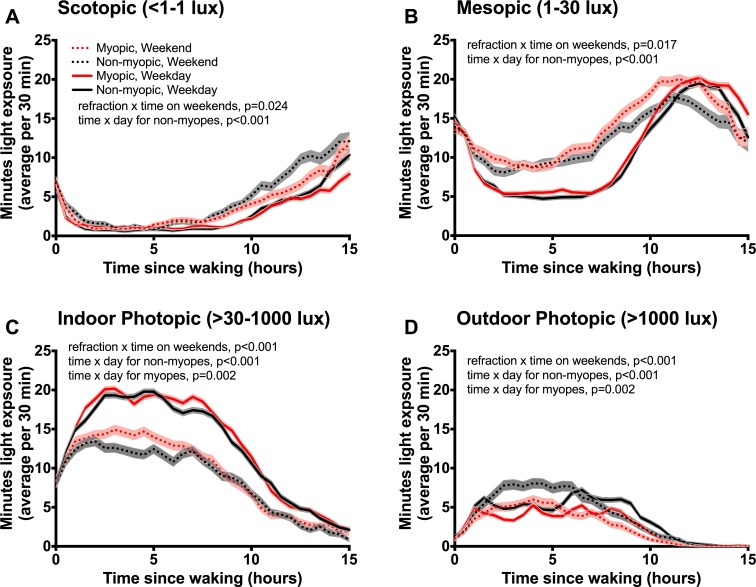
Patterns of daily light exposure across four intensity levels show differences in both dim and bright light as well as differences in behavior across the week. (A–D) Light exposure patterns of myopic and nonmyopic participants were mapped across the average 15 hours of awake time per day for each level of illuminance, then divided by weekday or weekend. (A) Recordings of scotopic light were observed immediately after waking and in the hours before bedtime. Myopic children received significantly less scotopic light during the weekend than nonmyopic children (ANCOVA, F_1,53_ = 5.38, P = 0.024). Scotopic light exposure patterns in nonmyopic children were higher on weekends compared with weekdays (F_1,53_ = 16.58, P < 0.001). (B) Mesopic light peaks in the evening then drops off approaching the average bedtime. Nonmyopic children spend more time in mesopic light on weekends compared with weekdays (F_1,53_ = 16.91, P < 0.001). On weekends, myopic children generally spend more time in mesopic light than nonmyopic children (F_1,53_ = 6.09, P = 0.017). (C) For each refractive group, exposure to indoor photopic light was significantly higher on weekdays compared with weekends (myopic children: F_1,53_ = 10.90, P = 0.002; nonmyopic children: F_1,53_ = 47.10, P < 0.001). On weekends, myopic children have significantly more indoor photopic light exposure (F_1,53_ = 14.32, P < 0.001). (D) Outdoor photopic light was highest in midday. Both myopic and nonmyopic children received more outdoor photopic light on weekends than on weekdays (myopic: F_1,53_ = 8.41, P = 0.002; nonmyopic: F_1,53_ = 20.39, P < 0.001). On weekends, nonmyopic participants have significantly more outdoor photopic exposure than myopic participants (F_1,53_ = 60.76, P < 0.001). P values shown in graphs represent significant interaction effects. Black lines represent nonmyopic children, red lines represent myopic children, and dashed lines represent weekends; data shown as mean ± SEM minutes of all subjects (n = 40/group) in bins of 30 minutes.

We examined the periods of day when exposure to each light level occurs for myopic and nonmyopic children and found significant differences in all light levels across the week and with refractive status (Fig. 1). The pattern of light exposure throughout the day was different for each of the four light levels. Scotopic light exposure primarily occurred in the hours before bed and immediately after waking, discrediting the possibility that this time is a false recording from the Actiwatch being covered by clothes or other items throughout the day. Myopic children received significantly less scotopic light during weekends than nonmyopic children (ANCOVA, *F*_1,53_ = 5.38, *P* = 0.024; Fig. 1A), with exposure during evening hours showing the largest differences (approximately 3 hours before falling asleep). In addition, scotopic light exposure for nonmyopic children was increased on weekends compared with weekdays (*F*_1,53_ = 16.58, *P* < 0.001). Nonmyopic children spent less time in mesopic light on weekdays than on weekends (*F*_1,53_ = 16.91, *P* < 0.001; Fig. 1B). However, on weekends myopic children generally spent more time in mesopic light than nonmyopic children (*F*_1,53_ = 6.09, *P* = 0.017). Indoor photopic light was increased in both groups on weekdays compared with weekends (myopic: *F*_1,53_ = 10.90, *P* = 0.002; nonmyopic: *F*_1,53_ = 47.10, *P* < 0.001; Fig. 1C). Outdoor photopic light was most prevalent in the middle of the day, corresponding with breaks in the school day or after school. Consistent with previously published findings, myopic children were exposed to less outdoor photopic light on weekends than nonmyopic children (ANCOVA, *F*_1,53_ = 60.76, *P* < 0.001; Fig. 1D).

**Figure 2:**
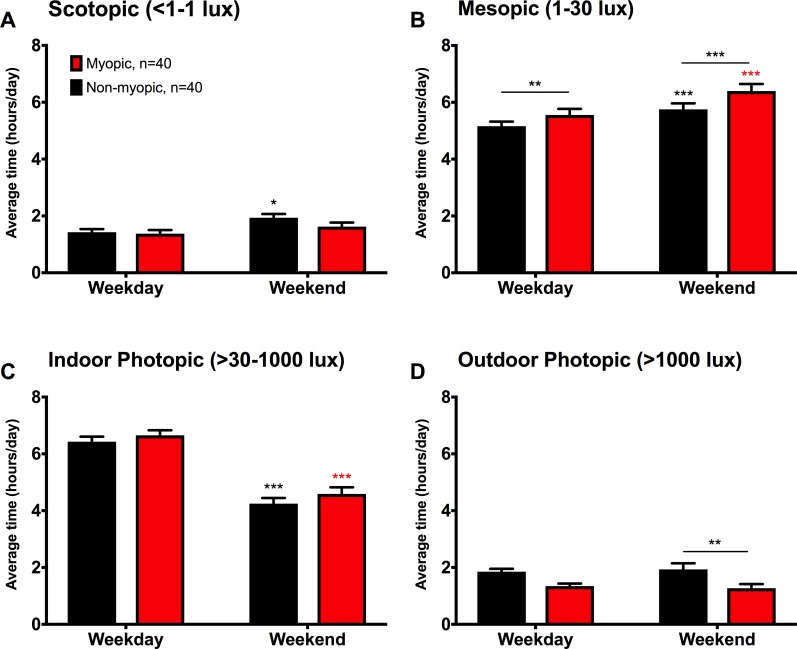
Myopic children spend less time in outdoor photopic and more time in mesopic light. Recordings of light intensity exposure during all waking hours were binned into four light levels and compared across myopic (red) and nonmyopic (black) children for weekdays and weekends. Data shown are the mean ± SEM of time spent in each light level per 15-hour awake period. (A) Nonmyopic children spend more time in scotopic light during the weekend compared with the weekdays (P = 0.026). (B) On weekdays and weekends, myopic children spend more time in mesopic light than nonmyopic children (P = 0.001, P < 0.001). Both myopic and nonmyopic children spend more time in mesopic light on weekends compared with weekdays (P < 0.001). (C) Both groups spend less time in indoor photopic light on weekends (myopic: P < 0.001; nonmyopic: P < 0.001). (D) On weekends, myopic children spent significantly less time in outdoor photopic light (P = 0.008). Asterisks above lines represent differences in refractive status groups, asterisks directly above bars represent differences across days, *P < 0.05, **P < 0.01, ***P < 0.001.

We also found a significant difference between myopic and nonmyopic children in the average daily light exposure in each light level summed across waking hours (multivariate ANOVA *F*_4,77_ = 3.87, *P* = 0.006; Wilk's Λ = 0.83, partial η2 = 0.17; Fig. 2). During weekdays, myopic children spend significantly more time in mesopic light than nonmyopic children (myopic: 5.56 ± 0.22 hours, nonmyopic: 5.16 ± 0.16 hours, *P* = 0.001; Fig. 2B). The differences between myopic and nonmyopic children did not reach significance for the other light levels during weekdays. However, there was a trend for myopic children spending less time than nonmyopic children in outdoor photopic light (1.35 ± 0.09 hours of waking time versus 1.85 ± 0.10 hours, *P* = 0.08; Fig. 2D). On weekends, myopic children spend significantly more time in mesopic light (myopic: 6.40 ± 0.25 hours, nonmyopic: 5.75 ± 0.21 hours, *P* < 0.001; Fig. 2B) and less time in outdoor photopic light than nonmyopic children (myopic: 1.27 ± 0.15 hours, nonmyopic: 1.93 ± 0.21 hours, *P* = 0.008; Fig. 2D). The exposure to each light level was also different between weekdays and weekends. Both myopic and nonmyopic children spend more time in mesopic light on weekends compared with weekdays (myopic, *P* < 0.0005; nonmyopic, *P* < 0.0005; Fig. 2B) and less time in indoor photopic (myopic, *P* < 0.0005; nonmyopic, *P* < 0.0005; Fig. 2C). However, only nonmyopic children spend more time in scotopic light on weekends compared with weekdays (*P* = 0.026; Fig. 2A). Furthermore, the additional amount of time nonmyopic children spent in scotopic light on the weekends was less than the additional amount of time spent in outdoor photopic light. Thus, nonmyopic children are spending more time in dim light outside of school hours.

**Figure 3:**
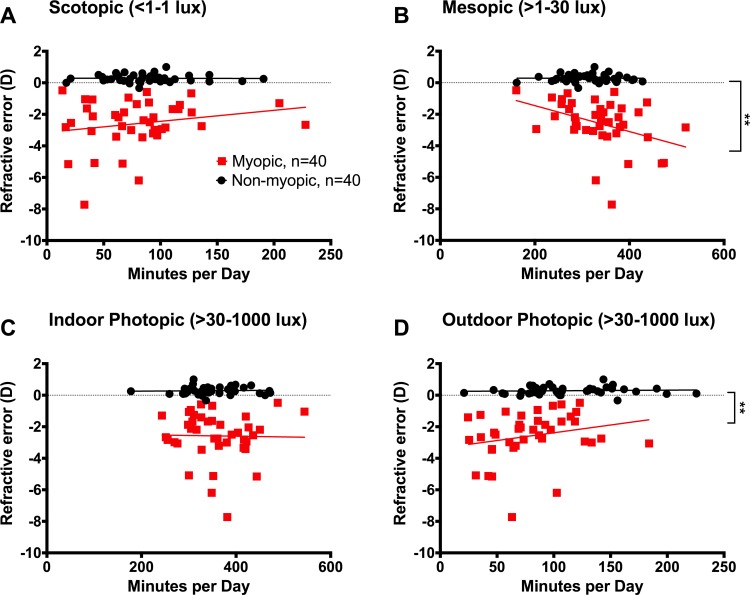
Significant correlation of mesopic and outdoor photopic light exposure and refractive errors of myopic children. (A) Time spent in scotopic light was not significantly associated with refractive error in nonmyopic children (black) or myopic children (red). (B) Increased time in mesopic light was significantly correlated with more severe myopia (R = −0.46, P = 0.002). (C) Indoor photopic light exposure times were similar in myopic and nonmyopic children and were not significantly associated with refractive error. (D) The time participants spent in outdoor photopic light was significantly associated with the refractive error of myopic children (R = 0.33, P = 0.005). Each point represents an individual participant.

An important consideration when developing recommendations for light exposure to prevent myopia is the relationship between the amount of time spent in different light levels and refractive error. The average of the initial refractive error measurements at the beginning of the study and the refractive measurement collected 1 year later was compared with the amount of time the children spent in each light intensity bin during the day (Fig. 3). No significant association between light and refractive error was found for the nonmyopic children across all four light bins, including nonmyopic children who spend little time in outdoor photopic light. In this data set, nonmyopic children had a very small range of refractive error, but the range of time in each light level matched that of the myopic children. Lower daily outdoor photopic light exposure was significantly correlated with more myopic refractive errors in myopic children (*R* = 0.33, *P* = 0.005; Fig. 3D). The correlations between time in both scotopic and outdoor photopic light and refractive error showed similar patterns (scotopic: slope: 0.007, *R* = 0.10, *P* = 0.43; photopic: slope: 0.009, *R* = 0.33, *P* = 0.005). As expected, myopic children showed a significant negative correlation for time in mesopic light (*R* = −0.46, *P* = 0.002) such that myopic children who spent more time in mesopic light had more severe myopia. Indoor photopic light had no relationship to refractive error in myopic children or nonmyopic children.

## Discussion

The data presented here demonstrate that myopic children spent less time in both scotopic and outdoor photopic light conditions compared with nonmyopic children. Although the association between less bright light exposure and myopia was expected based on previously reported studies in both human and animal models, a novel finding here was the significantly greater scotopic light exposure in the nonmyopic children. This suggests a potentially protective effect of both dim and bright light exposure in myopia development and potential myopiagenic effects of mesopic and indoor photopic light. Here, children spent more time in dim light levels than we had hypothesized, roughly equal to the amount of time spent in outdoor photopic light. Therefore, the potential of dim light as a prevention technique in myopia should be considered in further studies.

The retinal signaling mechanisms that would underlie the protection of myopia by bright and dim light are likely different given the differences in photoreceptor activation under such a broad range of illumination. Previously, no studies of human light exposure and myopia have directed attention to rod-dominated light levels. Here, we find that dim light, and potentially rod signaling mechanisms in the retina, could be playing a role in human myopia development, as shown in animal studies of myopia (Landis E. *IOVS* 2015;56:ARVO E-Abstract 2152).^29^ Cones, and potentially melanopsin photoreceptors, would be stimulated under bright light^46^ and may initiate signaling cascades that play a role in myopia prevention in bright light.^47,48^ Most investigations of time outdoors and the impact of light on refractive error in animal models have shown that increased dopamine activity triggered by bright light protects from induced myopia.^49^ This mechanism might explain how children who spend more time in bright light are protected from myopia. Potential protection from myopia under dim, scotopic light is likely through a different retinal signaling mechanism. Whether this mechanism also uses dopamine signaling is unclear but possible given the demonstrated connection between rod photoreceptors and dopamine release.^50,51^

The daily patterns of light exposure on both weekdays and weekends were similar across each of the light levels between myopic and nonmyopic children, even though the amount of time in each light level differed. One possible explanation for increased scotopic light at night could be the use of electronic devices before bed. Studies indicate that the use of tablets and cell phones before bed is very common in teenage children and that scotopic light from these devices could disrupt the circadian rhythms of participants.^52,53^ However, the lack of differences in the general daily patterns of light exposure between refractive groups along with the lack of significant differences in awake/sleep behavior would suggest that it is unlikely that there were differences in circadian rhythms associated with refractive error in this study. There is increasing evidence from animal models that circadian rhythms may play a role in myopia.^54^ Circadian rhythm genes have been implicated in myopia and the chick model has shown that the timing of both light exposure and lens defocus are important factors in myopia development.^55–59^ It is possible therefore that any changes in circadian rhythms that may lead to refractive error in humans may be subtler than what could be detected by the measurement methods and sample of subjects examined in the current study. It is also possible that myopic children experienced altered circadian rhythms at an age outside of the study dates.

The analysis used here differed in two ways from the analysis originally published: the number of children included in the final analysis differs from the Read et al.^16^ analysis and the waking hours were determined by activity recordings from the Actiwatch, whereas the original ROAM study analyzed readings from 6:00 AM to 6:00 PM. Although these changes made slight differences in the mean time of light exposure, the major finding that myopic children spend significantly less time in light >1000 lux than nonmyopic children was replicated. A limitation of the study is the position of the Actiwatch on the wrist instead of near the eye; however, previous reports of wrist-worn light sensor recordings have correlated strongly to eye-level light sensors except in late evening and at night, when the authors suspected bedding covered the wrist but not the eye-level sensor.^60^ This potential underestimation due to the participant's position in bed is a possible confounding factor in the findings presented here as well. However, the elimination of data collected while participants were sleeping, as measured through analysis of the physical activity data with validated algorithms,^42–44^ likely mitigates this complication. This limitation also raises the question of whether increased time spent in scotopic light was simply a reflection of decreased time spent in mesopic light, which would be protective against myopia. To investigate this possibility, total time spent in each light level was univariately correlated to time spent in scotopic light. This analysis showed that time spent in scotopic light was associated with less time in indoor photopic light only, indicating no significant connection between scotopic and mesopic light (Supplementary Fig. S2). The thresholds between the light levels used here were chosen based on similar studies in the case of higher intensity light exposure and on the ability of the Actiwatch to detect dim light in the case of the scotopic light threshold, set at 1 lux. The slight overestimation of light by the Actiwatch compared with a calibrated luxmeter for dim light levels (Supplementary Fig. S1) would indicate the scotopic threshold might be more stringent than is implied. The use of more accurate light sensors that provide better estimates of illuminance and spectral content of light at the plane of the eye in future analyses of dim light exposure is likely to provide a more comprehensive understanding of exposure patterns. Future analyses should also include cycloplegic refraction of study participants. The ROAM study included noncycloplegic refractions, which are known to be less reliable than cycloplegic, creating a risk of misclassification of refractive error groups (e.g., nonmyopic participants with undetected hyperopia). However, all myopic participants had previously been diagnosed with myopia and wore corrective lenses at the time of the study. Additionally, none of the participants changed between refractive groups during the first 12 months of the study, indicating consistency in their refractive classification over the course of the study.

Another limitation of this study is the relatively small sample size. With a larger population of children, it is possible that the differences in time spent under different levels of light would increase. Larger studies could aim to explore the behaviors of younger children; here, participants were between ages 10 and 15, and many were already myopic. By investigating light exposure in younger children, we might be able to determine what type of light exposure and signaling in the retina precedes the development of myopia.

We recommend that future studies on light exposure during refractive development in childhood include an analysis of dim light, especially in studies of younger children with larger populations. Because the findings reported here do not directly assess the effectiveness of light to prevent myopia through intervention, future studies may also be designed to establish causation. Finally, only data from waking hours were analyzed here; however, it is possible that light exposure while sleeping could also play a role in myopia development and progression.^61^

These findings, shown here for the first time in the human eye, are consistent with reports in animal models of myopia that have also demonstrated scotopic light exposure can be protective against myopia development. The results support a potentially key role for rod signaling in myopia development. Although the exact mechanisms underlying these findings remain unknown, early work in animal models could suggest different mechanisms across the light intensity range presented here depend on the specific pattern of photoreceptor activation. Therefore, these results provide a catalyst for future research to investigate the role of rod photoreceptors in myopia development.

## Supplementary Material

Supplement 1
