# Supplementary material for: Dim Light Exposure and Myopia in Children
Source: Invest Ophthalmol Vis Sci. 2018 Oct;59(12):4804–11. doi: 10.1167/iovs.18-24415 (PMC6181186; doi:10.1167/iovs.18-24415)
Supplement: Supplement 1 [file iovs-59-11-23_s01.pdf]

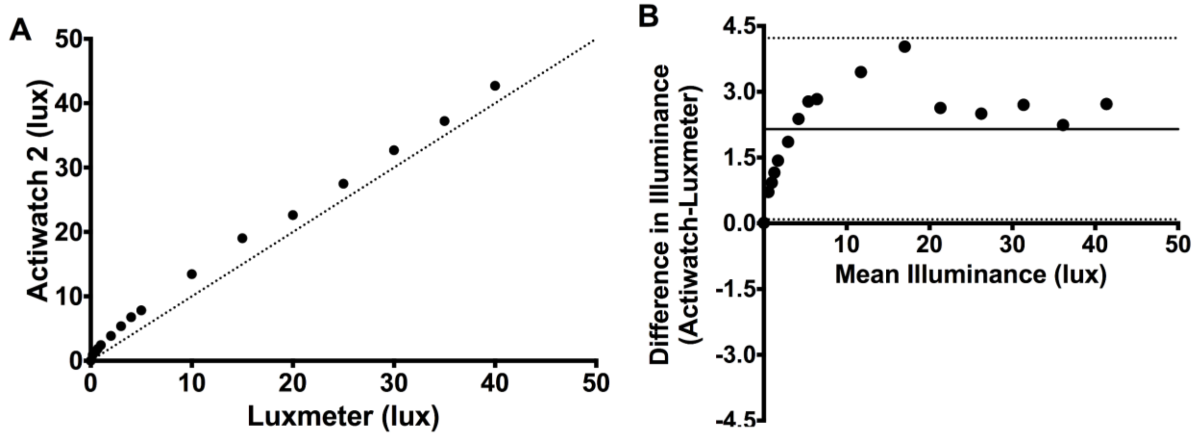

Supplementary Figure 1. Philips Actiwatch 2 accurately detects dim light levels. To determine the accuracy of the Actiwatch 2 at scotopic light levels the lux values of the Actiwatch were compared to a luxmeter (Extech HD450 Datalogging light meter; Extech Instruments, USA). The Actiwatch and luxmeter were placed in the same position in a room (both facing vertically upwards) and the ambient lights were changed through 16 different levels between 0 lux and 40 lux. The Actiwatch took 3 readings at each level which were averaged here. (A) There is a close correlation between the lux readings of the two light sensors (R^2=0.9958). Black dots represent recordings by Actiwatch and luxmeter at each brightness step, line represents an exact match of the values. (B) Differences between the Actiwatch and the luxmeter were found and compared to the mean readings between the two devices. This comparison shows that the Actiwatch overestimated lux by no more than 2.5 lux and that differences were greater at higher illuminance levels. Solid line represents the mean difference between Actiwatch and luxmeter readings, dotted lines represent upper and lower 95% limits of agreement.

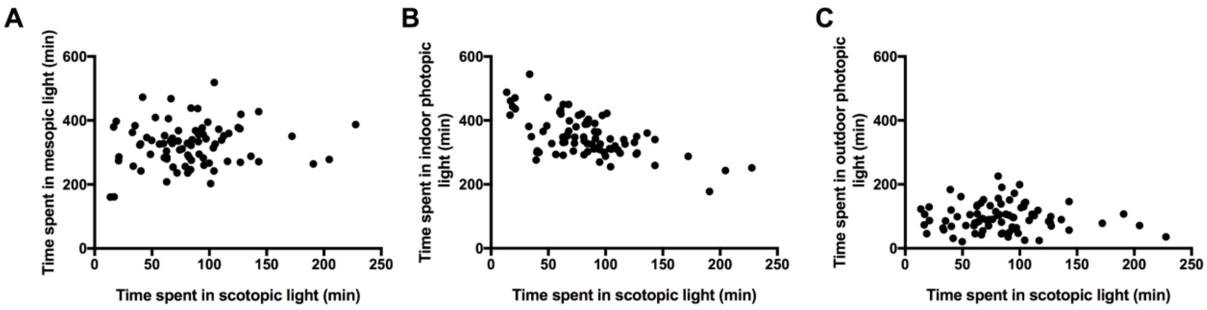

Supplementary Figure 2. Time spent in scotopic light is associated with time spent in indoor photopic light. To determine if effects of light exposure at one level might be driving any effects seen at other light levels, correlations between time spent in scotopic light and the other light levels were examined. While a significant correlation exists between scotopic light and indoor photopic (B; R=-0.646, p<0.0001), we found no relationship between scotopic exposure times and time in either mesopic (A) or outdoor photopic (C) light. For this analysis, Actiwatch data from all days were averaged for each subject and pooled.
